# Iron as a Key Mediator of Chronic Joint Damage: Insights from Hemophilic Arthropathy and Implications for Osteoarthritis

**DOI:** 10.3390/medicina62081515

**Published:** 2026-08-06

**Authors:** Michał Lubkowski, Zuzanna Leciej, Waldemar Pluta, Aleksandra Radecka, Anna Lubkowska

**Affiliations:** 1 Department of Functional Anatomy, Pomeranian Medical University, Al. Powstańców Wlkp. 72, 71-210 Szczecin, Poland; michal.lubkowski@pum.edu.pl; 2 Student Research at the Chair and Department of Functional Diagnostics and Physical Medicine, Pomeranian Medical University, Żołnierska 54, 71-210 Szczecin, Poland; 69943@student.pum.edu.pl; 3 Department of Functional Diagnostics and Physical Medicine, Pomeranian Medical University in Szczecin, Żołnierska 54, 71-210 Szczecin, Poland; waldemar.pluta@pum.edu.pl (W.P.); aleksandra.radecka@pum.edu.pl (A.R.)

**Keywords:** hemophilic arthropathy, hemophilia, hemarthrosis, intra-articular bleeding, iron metabolism, hemosiderin deposition, synovial inflammation, joint degeneration, cartilage destruction, knee osteoarthritis

## Abstract

*Background and Objectives*: Osteoarthritis (OA) is the most common degenerative joint disease and a leading cause of pain and disability worldwide. Increasing evidence indicates that dysregulated iron metabolism contributes to joint degeneration by promoting inflammation, oxidative stress, and cartilage damage. Hemophilic arthropathy (HA), a severe complication of hemophilia caused by recurrent hemarthroses, represents a unique model of iron-driven joint degeneration. This narrative review summarizes current evidence on the contribution of iron deposition to the pathogenesis of hemophilic arthropathy and discusses its relevance to knee osteoarthritis (KOA). *Materials and Methods*: The literature was reviewed using the PubMed, Scopus, and Web of Science databases, focusing on published studies addressing iron deposition, synovial inflammation, angiogenesis, oxidative stress, cartilage degeneration, subchondral bone remodeling, and dysregulated iron metabolism. *Results*: Current evidence indicates that iron stored primarily as hemosiderin promotes persistent synovial inflammation and progressive joint destruction. Several pathological pathways associated with iron accumulation, including chronic low-grade inflammation, extracellular matrix degradation, and altered bone remodeling, are shared by HA and KOA. *Conclusions*: These findings suggest that iron-mediated mechanisms may provide important insights into OA pathogenesis and support further investigation of iron as a potential diagnostic and therapeutic target in degenerative joint diseases.

## 1. Introduction

Recent experimental and clinical studies indicate that disturbances in iron homeostasis contribute significantly to the pathogenesis of osteoarthritis (OA), including knee osteoarthritis (KOA). Although iron is essential for numerous physiological processes, its excessive accumulation promotes the formation of reactive oxygen species (ROS) through Fenton chemistry, leading to oxidative stress, cellular dysfunction, and tissue damage. Within the osteoarthritic joint, iron overload has been implicated in cartilage degradation, synovial inflammation, and subchondral bone remodeling, all of which contribute to disease initiation and progression [1,2,3].

The association between iron dysregulation and OA is further supported by observations in patients with Hereditary Hemochromatosis (HH), most commonly caused by mutations in the *HFE* gene. Hemochromatosis-associated arthropathy is characterized by early onset and progressive joint degeneration, often showing limited response to iron-depletion therapy once structural damage has occurred [4,5,6].

Insight into the role of iron in joint degeneration has largely been derived from studies of hemophilic arthropathy (HA), which represents a unique human model of chronic intra-articular iron exposure. Recurrent hemarthroses result in repeated exposure of synovial and cartilaginous tissues to blood-derived iron, providing a natural setting to study iron-mediated joint damage. Hemophilia A and B, caused by deficiencies of coagulation factors VIII and IX, respectively, remain major inherited bleeding disorders associated with chronic musculoskeletal morbidity despite advances in prophylaxis. The knee, ankle, and elbow are most frequently affected, and recurrent bleeding episodes lead to progressive joint destruction and HA development [7,8].

Current evidence indicates that iron released from hemoglobin during erythrocyte breakdown is a key driver of joint pathology. Recurrent hemarthroses lead to progressive iron deposition within the synovium and periarticular tissues, triggering oxidative stress, chronic inflammation, and extracellular matrix degradation [9,10]. The resulting molecular and cellular pathways closely resemble those observed in OA, including synovial inflammation, cytokine overproduction, chondrocyte dysfunction, oxidative stress, and subchondral bone remodeling. These overlaps support the concept that dysregulated iron metabolism may represent a shared pathogenic mechanism in both conditions. Several recent studies have shown that iron accumulation enhances ROS production, amplifies inflammatory signaling, and accelerates cartilage degradation [11,12]. In addition, iron overload has been linked to chondrocyte senescence, mitochondrial dysfunction, and ferroptosis, an iron-dependent form of regulated cell death increasingly recognized in OA pathogenesis [2,13]. Elevated iron levels in synovial tissue and cartilage have also been associated with more severe structural damage in OA, further supporting its pathogenic relevance. Although growing experimental and clinical evidence supports a central role of iron dysregulation in joint degeneration, several iron-driven molecular pathways remain incompletely characterized. Accordingly, this review summarizes the current understanding of these proposed mechanisms while acknowledging that some pathways are still under investigation. Given the pronounced iron overload following recurrent hemarthroses, hemophilic arthropathy represents a valuable human model for studying iron-driven joint degeneration. HA exhibits both inflammatory and degenerative features, combining characteristics of rheumatoid arthritis and osteoarthritis. A deeper understanding of iron-mediated mechanisms in hemophilia may therefore provide novel insights into OA pathogenesis and identify potential therapeutic targets applicable to both conditions.

The aim of this narrative review is to summarize available knowledge regarding iron deposition in hemophilic joints, its role in synovial inflammation and joint degeneration, and its diagnostic and therapeutic implications, with particular emphasis on similarities between hemophilic arthropathy and knee osteoarthritis.

## 2. Materials and Methods

A comprehensive literature search was conducted using the PubMed, Scopus, and Web of Science databases. Original research articles, review papers, in vitro and animal studies, and clinical investigations published in English between 2015 and 2026 were screened for relevance. Earlier publications were also considered when they provided essential foundational evidence or clinically relevant information directly related to the mechanisms discussed in this review. The literature search was performed using combinations of the following keywords: “hemophilic arthropathy,” “hemophilia,” “osteoarthritis,” “knee osteoarthritis,” “iron metabolism,” “iron overload,” “iron deposition,” “hemosiderin,” “oxidative stress,” “reactive oxygen species,” “ferroptosis,” “synovitis,” “cartilage degeneration,” “subchondral bone,” and “inflammation.” The search focused on studies investigating the role of iron metabolism, oxidative stress, inflammation, ferroptosis, and iron-related molecular pathways in the pathogenesis of hemophilic arthropathy and osteoarthritis. Studies were selected based on their scientific quality and relevance to the objectives of this review. Particular emphasis was placed on molecular and cellular mechanisms underlying hemosiderin deposition, synovial inflammation and hyperplasia, angiogenesis, oxidative stress, ferroptosis, and cartilage and subchondral bone degeneration. When relevant, studies describing pathophysiological and clinical similarities between hemophilic arthropathy and osteoarthritis, particularly knee osteoarthritis, were also included. Additional relevant publications were identified through manual screening of the reference lists of selected articles.

## 3. Iron Accumulation in Joints: Pathophysiological Overview

### 3.1. Hemarthrosis as the Main Driver of Intra-Articular Iron Overload

Recurrent joint bleeding is the primary source of pathological iron accumulation within the synovial microenvironment of patients with hemophilia. During intra-articular bleeding, large numbers of erythrocytes enter the joint cavity. Under physiological conditions, blood degradation products are efficiently cleared by synovial macrophages; however, in hemophilia, repeated hemarthroses exceed this clearance capacity, resulting in progressive accumulation of hemoglobin, heme, and iron within joint tissues [14].

Importantly, contemporary imaging studies indicate that both clinically evident hemarthroses and subclinical bleeding episodes contribute to joint pathology. Magnetic resonance imaging (MRI) and musculoskeletal ultrasound have demonstrated synovial abnormalities and hemosiderin deposition even in joints without a history of overt bleeding, suggesting that repeated microhemorrhages may contribute significantly to disease progression [15,16]. Following erythrocyte lysis, hemoglobin and heme are internalized by synovial macrophages and fibroblast-like synoviocytes, where heme degradation releases free ferrous iron (Fe^2+^). Although intracellular iron is normally stored within ferritin complexes, recurrent bleeding can overwhelm local iron-handling mechanisms, leading to hemosiderin accumulation within the synovium. Due to limited iron-clearance capacity, repeated minor bleeding events may result in long-term iron deposition and tissue injury [17].

Comparable alterations in iron homeostasis have also been described in osteoarthritis (OA), where increased iron levels in synovial fluid and articular cartilage correlate with disease severity and structural abnormalities. Although the initiating factors differ between hemophilic arthropathy and OA, both conditions share downstream pathways involving oxidative imbalance, inflammation, extracellular matrix degradation, and tissue remodeling. These similarities support the role of hemophilic arthropathy as a clinically relevant model for investigating iron-mediated osteoarthritic changes [1,2,3,13,18].

### 3.2. Synovial Iron Deposition and Hemosiderin Accumulation: Mechanistic Pathways

Synovial macrophages (type A synoviocytes) represent the primary cellular component responsible for the uptake and sequestration of erythrocyte-derived products following intra-articular bleeding. These cells internalize hemoglobin and erythrocytes through scavenger receptors, including CD163 (hemoglobin–haptoglobin receptor) and CD64 [19].

During hemoglobin degradation, heme oxygenase-1 (HO-1) releases ferrous iron (Fe^2+^). Under physiological conditions, iron is safely stored within ferritin complexes; however, recurrent or chronic hemarthrosis exceeds this buffering capacity, resulting in persistent hemosiderin accumulation. Iron-loaded macrophages remain within the synovium as a continuous source of redox-active iron. Animal studies have confirmed that delayed clearance of these cells is associated with sustained iron deposition, impaired lymphangiogenesis, and chronic inflammatory activation [20,21].

Histologically, chronic synovial iron overload is characterized by intracellular and extracellular hemosiderin deposits detectable by Perls’ Prussian blue staining. This finding represents a hallmark of repeated blood exposure and reflects prolonged oxidative stress within the joint environment. Iron accumulation has also been identified in fibroblast-like synoviocytes, contributing to synovial hypertrophy and pathological tissue remodeling [21,22].

Advanced imaging approaches have further demonstrated an association between hemosiderin burden and hemophilic arthropathy severity. Iron-sensitive MRI sequences enable detection of synovial iron deposits, including in subclinical disease. Quantitative susceptibility mapping (QSM) provides a non-invasive approach for estimating tissue iron content through measurement of magnetic susceptibility changes caused by paramagnetic iron compounds. Recent studies suggest that QSM parameters correlate with synovial iron burden and structural joint damage, supporting their potential utility for disease monitoring and evaluation of therapeutic responses [17,23].

### 3.3. Iron as a Trigger of Synovial Inflammation: Cellular and Molecular Mechanisms

Accumulated iron is increasingly recognized not as a passive consequence of joint bleeding but as an active mediator linking recurrent hemarthrosis to chronic synovitis and structural joint damage. The synovium (SM) functions as a semi-permeable, size-selective membrane regulating molecular exchange within the joint while maintaining synovial fluid composition essential for cartilage homeostasis [24]. It consists mainly of fibroblast-like synoviocytes (FLSs) embedded in an extracellular matrix (ECM) containing hyaluronic acid collagen (including types I, III, IV, V, and VI), and proteoglycans. Under physiological conditions, the synovial lining contains only a limited number of macrophages and inflammatory cells [25,26]. However, early-stage OA is frequently associated with synovial thickening and inflammatory cell infiltration. Iron contributes to this process through oxidative stress, lipid peroxidation, and persistent activation of inflammatory pathways.

A central mechanism underlying iron-associated synovial inflammation is the generation of reactive oxygen species (ROS). Redox-active ferrous iron (Fe^2+^) catalyzes the conversion of hydrogen peroxide into highly reactive hydroxyl radicals through the Fenton reaction, resulting in oxidative damage to cellular membranes, proteins, and nucleic acids within synovial and cartilage tissues. Iron-induced ROS also promote lipid peroxidation and impair glutathione peroxidase 4 (GPX4) activity, a key regulator involved in ferroptosis and osteoarthritis progression [27]. Oxidative stress and lipid peroxidation products activate NF-κB (Nuclear Factor kappa-light-chain-enhancer of activated B cells) signaling, inducing the production of pro-inflammatory cytokines, including interleukin-1β (IL-1β), interleukin-6 (IL-6), and tumor necrosis factor-α (TNF-α).

These pathways enhance innate immune activation and promote the release of damage-associated molecular patterns (DAMPs), thereby sustaining chronic synovial inflammation. In parallel, iron exposure increases the expression of matrix metalloproteinases (MMPs) and aggrecanases, accelerating extracellular matrix degradation and cartilage damage. Collectively, these mechanisms link recurrent hemarthrosis with chronic synovitis and tissue deterioration. Loss of extracellular matrix integrity, impaired cartilage biomechanics, and persistent inflammatory activation contribute to hemophilic arthropathy and share common features with OA, including cartilage loss, subchondral bone remodeling, and impaired joint function [28,29,30].

In addition to macrophages, fibroblast-like synoviocytes acquire a pro-inflammatory and invasive phenotype in response to iron- and hemoglobin-derived stimuli. Iron-related oxidative stress promotes synovial activation characterized by increased proliferation, reduced susceptibility to apoptosis, and enhanced production of inflammatory mediators. In vitro studies demonstrated that FLS stimulated with TNF-α for 48 h exhibited significantly increased IL-6 secretion, confirming that TNF-α directly induces a pro-inflammatory response in these cells [31]. Activated synoviocytes further contribute to tissue damage by increasing the expression of matrix-degrading enzymes, including MMP-1, MMP-3, MMP-13, and ADAMTS aggrecanases [32,33].

Similar iron-associated inflammatory and degenerative processes have been described in OA, where iron accumulation correlates with synovial inflammation, chondrocyte dysfunction, and extracellular matrix degradation. Increased iron crystal deposition in synovial-lining cells and elevated iron concentrations in synovial fluid have been reported in knee OA, together with higher serum ferritin levels in affected patients [34]. Furthermore, an arthroscopic scoring study in knee OA demonstrated that synovitis severity is strongly associated with cartilage destruction, suggesting its potential value in predicting disease progression [35].

These findings indicate that iron-related pathways may represent an important mechanistic link between hemophilic arthropathy and OA and support investigation of ferroptosis inhibition as a potential disease-modifying strategy. Although HA is initiated by recurrent intra-articular bleeding and OA is primarily driven by degenerative processes, both conditions converge on common downstream pathways involving oxidative stress, NF-κB activation, and extracellular matrix breakdown. Thus, iron may represent a shared mediator contributing to chronic joint pathology.

Overall, current evidence supports the concept that iron functions as an active regulator of persistent synovial inflammation rather than merely a consequence of recurrent bleeding. By promoting oxidative imbalance, inflammatory signaling, and matrix degradation, iron contributes to tissue damage in both hemophilic arthropathy and osteoarthritis. These interconnected processes provide a mechanistic basis for the development of hypertrophic synovitis and sustained NF-κB activation discussed in the following section.

### 3.4. Iron-Induced Hypertrophic Synovitis and NF-κB-Mediated Inflammation

The synovium is a highly specialized, multifunctional structure lining the inner surface of the articular capsule that maintains joint homeostasis. This function is primarily mediated by fibroblast-like synoviocytes (FLSs), which regulate the production of extracellular matrix (ECM) components and synovial fluid constituents. In pathological conditions, FLSs also contribute to the production of matrix-degrading enzymes, including matrix metalloproteinases (MMPs) and aggrecanases, which drive joint destruction in osteoarthritis (OA). Synovial hypertrophy in rheumatoid arthritis (RA) is typically driven by FLS proliferation, neovascularization, and infiltration of immune cells. FLS activation in RA is accompanied by multiple associated alterations in intracellular metabolic pathways, typified by alterations in gluconeogenesis, lipolysis, and amino acid metabolism [36]. In contrast, synovial lesions in hemophilic arthropathy are more strongly associated with iron deposition than with autoimmune-driven inflammation or primary degenerative processes. Bleeding into the joints can trigger the release of IL-1β, IL-6, C-X-C motif chemokine ligand 1 (CXCL1) and CCL2 (C-C Motif Chemokine Ligand 2), contributing to joint inflammation and pain. Synovial tissues from patients with HA exhibit significantly higher expression of iron-regulatory proteins, including ferroportin (FPN), CD163, and heme carrier protein 1 (HCP-1), compared with RA, OA, and healthy controls [37,38].

Intracellular iron accumulation has been shown to promote synovial cell proliferation and survival through modulation of cell-cycle regulatory pathways, including upregulation of MYC (MYC proto-oncogene, bHLH transcription factor) and MDM2 (MDM2 proto-oncogene), as well as regulation of p21 (Cyclin-Dependent Kinase Inhibitor 1A) expression. As a key regulator of the G1/S checkpoint, p21 contributes to synovial fibroblast proliferation dynamics and cellular stress responses. In parallel, p53 (Tumor Protein p53)-dependent induction of p21 in fibroblast-like synoviocytes may result in cell-cycle arrest rather than apoptosis, thereby promoting synovial tissue expansion and a hyperplastic phenotype [25,39]. Iron-induced oxidative stress further amplifies these effects through direct activation of NF-κB- and MAPK (Mitogen-Activated Protein Kinase)-dependent signaling pathways, creating a pro-inflammatory and pro-proliferative synovial microenvironment. Activation of NF-κB leads to transcriptional upregulation of multiple inflammatory and catabolic mediators, including TNF-α, interferon-γ, IL-1β, IL-6, cyclooxygenase-2 (COX-2), and matrix metalloproteinases (MMPs). These mediators collectively enhance synovial inflammation and accelerate degradation of extracellular matrix components, particularly collagen type II and aggrecan. Sustained NF-κB activation represents a critical molecular link between iron-induced synovitis and progressive cartilage destruction, contributing to hemophilic arthropathy and OA-like joint degeneration [40,41,42]. Therefore, persistent iron deposition following recurrent overt or subclinical hemarthroses promotes pathological synovial hypertrophy, a hallmark feature of hemophilic arthropathy [14,43]. The hypertrophic synovium subsequently becomes a major source of inflammatory mediators, angiogenic factors, and matrix-degrading enzymes, thereby sustaining chronic synovitis, recurrent bleeding, and progressive joint destruction.

### 3.5. Neoangiogenesis and the Vicious Circle of Bleeding

Pathological synovial hyperplasia and iron accumulation act synergistically to promote neovascularization, as increased metabolic demands of hypertrophic tissue and iron-associated oxidative stress stimulate pathological angiogenesis in chronic hemophilic synovitis. Proliferating synovium releases chemotactic and angiogenic mediators, including vascular endothelial growth factor (VEGF), which promotes recruitment of endothelial cells (ECs) to sites of active vessel formation [44]. Oxidative stress and inflammatory cytokines further enhance VEGF expression, leading to the development of immature and fragile blood vessels within the synovial membrane.

The number of macrophages/monocytes, endothelial progenitor cells, and hematopoietic progenitor cells is increased in hemophilic synovial tissue compared with normal synovium. Macrophages contribute directly to neovascularization by releasing mediators that stimulate fibroblasts and endothelial cells to produce VEGF. In addition, lymphocytes and neutrophils promote angiogenic responses through secretion of pro-angiogenic factors, including VEGF and basic fibroblast growth factor (bFGF). These immune cells contribute to the initiation and amplification of angiogenic signaling within chronically inflamed tissues [45]. Increased VEGF expression has also been reported in rheumatoid arthritis (RA) and osteoarthritis (OA), supporting its role as a shared mediator of synovial angiogenesis across different joint diseases [31,46,47].

Newly formed vessels are characterized by increased permeability and mechanical fragility, making them susceptible to rupture and further intra-articular bleeding. Consequently, iron deposition, synovial inflammation, hyperplasia, and angiogenesis become interconnected in a self-perpetuating cycle that promotes chronic joint damage [7]. This pathological cascade can be summarized as follows:

hemarthrosis → iron deposition → synovitis → angiogenesis → recurrent hemarthrosis → increased iron accumulation

Angiogenesis also influences cartilage pathology by promoting chondrocyte hypertrophy, abnormal tissue growth, altered perfusion, and endochondral ossification. Synovectomy may interrupt this pathological cycle and remains an established therapeutic option in hemophilic arthropathy. Current approaches include radiosynovectomy, arthroscopic synovectomy, and open surgical synovectomy. By removing abnormal synovial tissue, these interventions reduce recurrent hemarthroses, iron deposition, and inflammatory activation in hemophilic arthropathy and may also provide symptomatic benefits in other chronic synovial disorders, including osteoarthritis [48,49,50,51].

### 3.6. Mechanisms of Iron-Driven NLRP3 Inflammasome Activation

Recent studies have demonstrated that iron-induced oxidative stress, mitochondrial dysfunction, and lysosomal destabilization can activate the NOD-like receptor family, pyrin domain-containing 3 (NLRP3) inflammasome [52]. This cytoplasmic multiprotein complex plays a central role in innate immunity by sensing cellular stress and damage-associated molecular patterns (DAMPs), as well as pathogen-associated molecular patterns (PAMPs) [53]. Mitochondrial dysfunction represents an important upstream amplifier of NLRP3 inflammasome activation in iron-overloaded joints. Excess intracellular Fe^2+^ promotes mitochondrial reactive oxygen species (mtROS) production, loss of mitochondrial membrane potential, and oxidative damage to mitochondrial components. These events collectively facilitate both priming and activation of the NLRP3 inflammasome, thereby linking iron-driven oxidative stress to innate immune signaling and sustained inflammation [54]. In this context, mitochondrial damage acts as a central hub integrating metabolic stress with inflammasome activation pathways. Mitochondrial damage further leads to the release of mitochondrial DNA (mtDNA) into the cytosol, which acts as a potent damage-associated molecular pattern (DAMP) capable of directly activating the NLRP3 inflammasome complex [55,56]. These mitochondrial signals promote NLRP3 oligomerization and recruitment of ASC (Apoptosis-Associated Speck-Like Protein Containing a CARD) and pro-caspase-1, resulting in caspase-1 activation and subsequent maturation of interleukin-1β and interleukin-18 (IL-18), thereby reinforcing synovial inflammation and linking metabolic stress to innate immune activation [54,57,58,59]. Upon activation, the NLRP3 inflammasome promotes recruitment and activation of caspase-1, which mediates the proteolytic maturation of pro-inflammatory cytokines interleukin-1β and interleukin-18. These cytokines are key regulators of synovial inflammation, promoting leukocyte infiltration, synovial cell activation, and production of catabolic mediators that drive progressive joint damage [14,60]. IL-1β further enhances vascular permeability, facilitates inflammatory cell recruitment into the synovium, and stimulates the production of matrix metalloproteinases (MMPs), thereby accelerating extracellular matrix degradation and cartilage destruction. In parallel, IL-1β amplifies synovial fibroblast activation and sustains the production of additional pro-inflammatory mediators, maintaining a self-perpetuating inflammatory microenvironment. Emerging evidence suggests that persistent activation of the ROS–NLRP3–IL-1β axis represents a key pathogenic mechanism in hemophilic arthropathy, even after the resolution of acute bleeding episodes. Iron-induced oxidative stress promotes continuous inflammasome activation and sustained IL-1β release, which overall drive chronic synovitis, extracellular matrix degradation, and progressive joint degeneration.

Thus, the ROS–NLRP3–IL-1β signaling pathway has emerged as a potential therapeutic target for preventing long-term joint damage in hemophilic arthropathy and related degenerative joint diseases [3,20,61,62].

### 3.7. Ferroptosis

Ferroptosis is an iron-dependent regulated form of cell death characterized by the accumulation of lipid peroxides within cellular membranes. Its execution is primarily determined by the balance between iron-catalyzed lipid oxidation and glutathione peroxidase 4 (GPX4)-dependent antioxidant defense systems [63,64]. At the molecular level, ferroptosis is driven by expansion of the labile iron pool, which increases the availability of redox-active Fe^2+^ and promotes lipid peroxidation in polyunsaturated phospholipids. This process is further amplified by lipoxygenase (ALOX)-mediated enzymatic oxidation, contributing to the formation of phospholipid hydroperoxides, particularly in phosphatidylethanolamine species [65,66]. Detoxification of lipid hydroperoxides is mainly mediated by the GPX4–glutathione (GSH) axis. GPX4 reduces lipid hydroperoxides to non-toxic lipid alcohols using GSH as a cofactor, thereby preventing propagation of lipid radical chain reactions. Impairment of GPX4 activity or depletion of GSH leads to uncontrolled lipid peroxidation and membrane damage, ultimately resulting in ferroptotic cell death. In iron-overloaded joint microenvironments, sustained elevation of intracellular Fe^2+^ shifts redox homeostasis toward lipid oxidation, exceeding GPX4-dependent antioxidant capacity [11,67,68,69]. This results in membrane instability and mitochondrial vulnerability, as both structures are highly sensitive to lipid peroxidation. Chondrocytes are particularly susceptible to ferroptotic injury due to limited regenerative capacity and dependence on redox homeostasis for maintenance of extracellular matrix integrity. Reduced GPX4 activity has been associated with impaired anabolic function and increased matrix degradation, linking ferroptosis to degenerative joint pathology [13,69,70,71]. Ferroptosis is additionally regulated by the cystine/glutamate antiporter system Xc^−^ (SLC7A11/SLC3A2), which controls intracellular glutathione synthesis. Downregulation of SLC7A11 under oxidative stress conditions reduces cystine uptake, thereby limiting GSH availability and further weakening GPX4 activity. In parallel, acyl-CoA synthetase long-chain family member 4 (ACSL4) promotes incorporation of polyunsaturated fatty acids into membrane phospholipids, increasing susceptibility to lipid peroxidation [63,72]. Taken together, the GPX4–System Xc^−^ axis represents a key redox checkpoint determining cellular susceptibility to iron-induced lipid peroxidation and ferroptotic injury in joint tissues [33,66].

Given the emerging role of ferroptosis in joint degeneration, pharmacological modulation of ferroptotic pathways has become a promising area of research. Experimental studies investigating ferroptosis inhibitors, iron chelators, GPX4 activators, and antioxidants have shown encouraging results in preclinical models, although further studies are needed to establish their safety, efficacy, and potential clinical application in hemophilic arthropathy and osteoarthritis.

### 3.8. Hepcidin–Ferroportin Axis in Local Iron Overload of the Synovium

In recent years, increasing attention has been given to the dysregulation of local iron homeostasis within the joint microenvironment. A central role in this process is played by the hepcidin–ferroportin axis, which regulates iron efflux at both systemic and tissue levels. Hepcidin is a peptide hormone primarily synthesized by hepatocytes; however, its local expression has also been demonstrated in immune cells, including synovial macrophages [73,74]. Hepcidin exerts its biological effect by binding to ferroportin (FPN), the only known cellular iron exporter, thereby inducing its internalization and lysosomal degradation. This process results in functional iron retention within cells and reduced iron efflux into the extracellular space [75]. During recurrent intra-articular bleeding, substantial amounts of heme-derived iron are phagocytosed by synovial macrophages, resulting in progressive intracellular iron accumulation [21,49]. Concurrently, chronic synovial inflammation promotes sustained production of interleukin-6 (IL-6), a key regulator of hepcidin expression. Elevated hepcidin levels induce the internalization and degradation of ferroportin, the only known cellular iron exporter, thereby reducing iron efflux and promoting intracellular iron retention in synovial macrophages [76].

Together, these mechanisms may establish a self-perpetuating cycle of iron sequestration, oxidative stress, and synovial inflammation, ultimately contributing to progressive joint damage in hemophilic arthropathy. Hence, synovial macrophages acquire a persistent iron-loaded phenotype that promotes redox imbalance, inflammasome activation, and sustained inflammatory signaling. Thus, the IL-6–hepcidin–ferroportin axis represents a key regulatory pathway linking inflammation with local iron accumulation and may contribute to the persistence of iron overload and chronic synovitis in hemophilic arthropathy.

### 3.9. Iron and Subchondral Bone Remodeling: Osteoclast Activation and Bone Resorption

Beyond synovial inflammation and cartilage degeneration, dysregulated iron metabolism contributes to structural alterations in subchondral bone, primarily through modulation of osteoclast differentiation and activity. Subchondral bone remodeling is a dynamic process in both hemophilic arthropathy and osteoarthritis, in which altered bone resorption precedes and accompanies cartilage degeneration. Iron overload promotes osteoclastogenesis by shifting the balance of the receptor activator of nuclear factor κB ligand (RANKL)/osteoprotegerin (OPG) axis toward increased RANKL expression and reduced OPG activity. This imbalance enhances osteoclast formation and activation, leading to excessive bone resorption, subchondral bone plate thinning, and structural weakening of the osteochondral unit [77,78,79].

At the cellular level, iron acts as a pro-osteoclastogenic factor by enhancing reactive oxygen species (ROS)-dependent signaling pathways required for osteoclast precursor differentiation. Osteoclastogenesis is highly sensitive to intracellular redox status, and iron-induced oxidative imbalance potentiates activation of NFATc1, the master transcription factor regulating osteoclast differentiation. This results in upregulation of osteoclast-specific genes, including tartrate-resistant acid phosphatase (TRAP), cathepsin K, and matrix metalloproteinase-9 (MMP-9), thereby increasing bone-resorptive activity [80,81]. In addition, iron-driven inflammatory signaling may further enhance osteoclast activity through cytokine-mediated upregulation of RANKL expression in osteoblasts and synovial fibroblasts. This establishes a functional coupling between synovial inflammation and subchondral bone resorption, thereby linking soft tissue pathology to structural bone damage [82,83]. In osteoarthritis, subchondral bone remodeling is characterized by early osteoclastic activation followed by aberrant bone formation and sclerosis. Similar alterations are observed in hemophilic arthropathy, where recurrent exposure to blood-derived iron may accelerate early osteoclast-mediated bone resorption, contributing to joint instability and progressive structural damage [1,2,84,85]. Overall, these findings support the concept that iron dysregulation may act as a shared upstream regulator of pathological bone remodeling in both hemophilic arthropathy and osteoarthritis.

**Figure 1:**
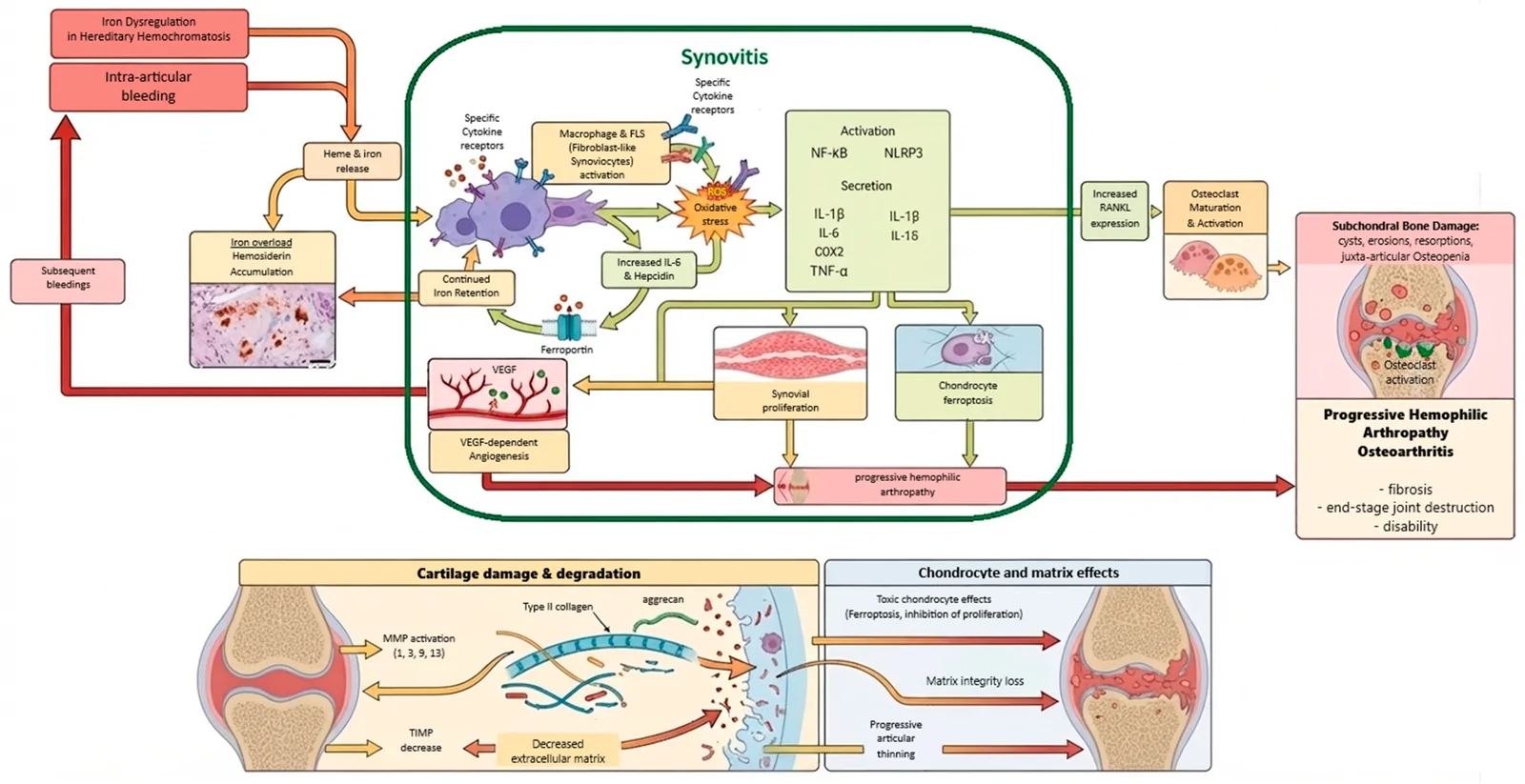
Iron-driven pathophysiological mechanisms underlying hemophilic arthropathy and osteoarthritis. The figure provides an integrated overview of the current understanding of the proposed iron-driven mechanisms involved in the pathogenesis of hemophilic arthropathy and osteoarthritis. Iron accumulation promotes oxidative stress, inflammation, synovial hypertrophy, cartilage degradation, subchondral bone remodeling, and ferroptosis, contributing to progressive joint damage. While several of these pathways are supported by experimental and clinical evidence, others remain incompletely understood and are still under investigation. The schematic summarizes the current evidence and proposed interactions between these interconnected mechanisms rather than depicting fully established causal pathways.

The comprehensive overview of the pathophysiological pathways linking intra-articular bleeding, iron dysregulation, synovial inflammation, and progressive joint damage in both hemophilic arthropathy and osteoarthritis is summarized in Figure 1.

## 4. Conclusions and Future Directions

Available evidence supports the concept that dysregulated iron metabolism represents a central pathogenic axis linking hemophilic arthropathy and osteoarthritis (OA). Although the initiating triggers of these conditions differ—recurrent intra-articular bleeding in hemophilia versus chronic mechanical, metabolic, and inflammatory stress in OA—both converge on shared downstream mechanisms characterized by synovial iron accumulation, oxidative stress, inflammasome activation, NF-κB signaling, extracellular matrix degradation, and subchondral bone remodeling. The recognition of iron as an active driver of joint degeneration rather than a passive by-product of tissue injury has reshaped current understanding of chronic arthropathies. Within both HA and OA, iron overload functions as a central amplifier of tissue injury by sustaining redox imbalance and promoting persistent activation of innate immune and stress-response pathways. This results in progressive dysfunction of synoviocytes, macrophages, chondrocytes, and osteoclast-lineage cells, ultimately contributing to synovial hypertrophy, cartilage degeneration, and structural joint failure. These convergent molecular features support the concept of an “iron-driven degenerative joint phenotype,” which may represent a shared pathogenic framework across distinct chronic arthropathies.

Despite significant progress, important gaps remain. The temporal dynamics of iron accumulation in early-stage OA and its causal contribution to disease initiation remain incompletely defined. Likewise, the relative roles of systemic iron dysregulation versus local intra-articular iron recycling require further clarification. The interplay between ferroptosis, inflammasome activation, and NF-κB-mediated inflammation also needs to be delineated in a stage-specific manner to better understand disease progression.

From a translational perspective, future research should focus on the development of validated biomarkers of intra-articular iron burden, including advanced imaging techniques such as quantitative susceptibility mapping (QSM), as well as synovial fluid and circulating iron-regulatory signatures. Therapeutic strategies targeting iron metabolism—including modulation of the hepcidin–ferroportin axis, iron chelation approaches, and ferroptosis inhibition—represent promising but still largely unexplored disease-modifying interventions. Whether such strategies can alter the natural history of OA or prevent progression of established hemophilic arthropathy remains an open and clinically relevant question.

Finally, hemophilic arthropathy represents a valuable human model of iron-driven joint degeneration and may provide key mechanistic insights applicable to OA.

A deeper integration of hematological and osteoarthritic research fields may facilitate the identification of shared therapeutic targets and clinically relevant biomarkers for chronic degenerative joint diseases. Future studies should test whether intra-articular iron accumulation can predict disease progression and whether targeting iron-regulatory pathways, including the hepcidin–ferroportin axis, iron chelation, or ferroptosis-related signalling, can modify disease outcomes rather than only alleviate symptoms. These hypotheses may provide a framework for translational research aimed at developing mechanism-based diagnostic and therapeutic approaches for hemophilic arthropathy and osteoarthritis.

## 5. Limitations

This narrative review is limited by the heterogeneity of included studies, encompassing in vitro, in vivo, imaging, and clinical data, which restricts direct comparison across methodologies. Most mechanistic evidence regarding ferroptosis, inflammasome activation, and NF-κB signaling is derived from preclinical models and requires further validation in human synovial tissues, particularly in osteoarthritis. Notably, evidence on iron dysregulation is more robust in hemophilic arthropathy than in osteoarthritis, which may bias mechanistic extrapolation. As a non-systematic review, no formal risk-of-bias assessment or quantitative synthesis was performed; therefore, findings should be interpreted as hypothesis-generating.

## Data Availability

No new data were created or analyzed in this study. Data sharing is not applicable to this article.

## Abbreviations

ACSL4	Acyl-CoA Synthetase Long-Chain Family Member 4
ASC	Apoptosis-Associated Speck-like Protein Containing a CARD
bFGF	Basic Fibroblast Growth Factor
CCL2	C-C Motif Chemokine Ligand 2
c-MYC	MYC proto-oncogene, bHLH transcription factor
COX-2	Cyclooxygenase-2
CXCL1	C-X-C Motif Chemokine Ligand 1DAMPs—Damage-Associated Molecular Patterns
EC	Endothelial Cell
ECM	Extracellular Matrix
FPN	Ferroportin
GPX4	Glutathione Peroxidase 4
HCP-1	Heme Carrier Protein 1
HO-1	Heme Oxygenase-1
HA	Hemophilic Arthropathy
IL-1β	Interleukin-1 beta
IL-6	Interleukin-6
IL-18	Interleukin-18
MAPK	Mitogen-Activated Protein Kinase
MDM2	pro-to-oncogene
MMP/MMPs	Matrix Metalloproteinase(s)
mtDNA	Mitochondrial DNA
mtROS	Mitochondrial Reactive Oxygen Species
NF-κB	Nuclear Factor kappa-light-chain-enhancer of activated B cells
NLRP3	NOD-Like Receptor Family Pyrin Domain Containing 3
NFATc1	Nuclear Factor of Activated T Cells 1
OA	Osteoarthritis
PAMPs	Pathogen-Associated Molecular Patterns
p21	Cyclin-Dependent Kinase Inhibitor 1A, CDKN1A
p53	Tumor Protein p53
QSM	Quantitative Susceptibility Mapping
RA	Rheumatoid Arthritis
RANKL	Receptor Activator of Nuclear Factor κB Ligand
ROS	Reactive Oxygen Species
SLC7A11	Solute Carrier Family 7 Member 11
SLC3A2	Solute Carrier Family 3 Member 2
SM	Synovial Membrane
TNF-α	Tumor Necrosis Factor alpha
TRAP	Tartrate-Resistant Acid Phosphatase
VEGF	Vascular Endothelial Growth Factor
